# Yiqi Huoxue Recipe Improves Heart Function through Inhibiting Apoptosis Related to Endoplasmic Reticulum Stress in Myocardial Infarction Model of Rats

**DOI:** 10.1155/2014/745919

**Published:** 2014-04-17

**Authors:** Li-Xia Lou, Ai-Ming Wu, Dong-Mei Zhang, Sheng-Xian Wu, Yong-Hong Gao, Bo Nie, Ming-Jing Zhao, Xi-Ying Lv, Qiu-Shuo Jin, Yi-Zhou Zhao, Shuo-Ren Wang, Li-Min Chai

**Affiliations:** Key Laboratory of Chinese Internal Medicine of Ministry of Education and Beijing, Dongzhimen Hospital Affiliated to Beijing University of Chinese Medicine, Beijing 100700, China

## Abstract

*Objective*. To explore the mechanism of cardioprotective effects of Chinese medicine, Yiqi Huoxue recipe, in rats with myocardial infarction- (MI-) induced heart failure. *Methods*. Male Sprague-Dawley rats underwent left anterior descending artery (LAD) ligation or sham operation. The surviving MI rats were divided randomly into three groups: MI (5 mL/kg/d NS by gavage), MI + Metoprolol Tartrate (MT) (12 mg/kg/d MT by gavage), and MI + Yiqi Huoxue (5 mL/kg recipe by gavage). And the sham operation rats were given 5 mL/kg/d normal saline. Treatments were given on the day following surgery for 4 weeks. Then rats were detected for heart structure and function by transthoracic echocardiography. Apoptosis in heart tissues was detected by TUNEL staining. To determine whether the endoplasmic reticulum (ER) stress response pathway is included in the cardioprotective function of the recipe, ER stress related proteins such as GRP78 and caspase-12 were examined. *Results*. Yiqi Huoxue recipe attenuated heart function injury, reversed histopathological damage, alleviated myocardial apoptosis and inhibited ER stress in MI rats. *Conclusion*. All the results suggest that Yiqi Huoxue recipe improves the injured heart function maybe through inhibition of ER stress response pathway, which is a promising target in therapy for heart failure.

## 1. Introduction 

Heart failure is a syndrome with high mortality and morbidity where cardiac function is inadequate to meet metabolic needs of the body. Heart failure is now one of the greatest public health problems [1]. Thus, the development of novel treatments for patients with cardiovascular diseases remains a major research priority. The most common cause of heart failure is coronary artery disease (CAD) and CAD related myocardial infarction (MI). Multiple factors are involved in the mechanism of heart failure progression, such as the activity of sympathetic nervous system. During the last few years there has been increasing evidence from human and animal models suggesting that apoptosis could be a key modulator especially in the transition from compensatory hypertrophy to heart failure [2, 3]. It is well known that the death receptor and mitochondrial apoptotic pathways are the two main apoptotic pathways leading to cardiomyocytes death in heart failure. More and more researches have confirmed that endoplasmic reticulum (ER) is a primary target in various acute disorders and has been suggested as the third subcellular compartment implicated in apoptotic pathway [4]. Recent studies have revealed that ER stress and ER related apoptosis were important mechanisms included in heart failure progression [5, 6].

Oxidative stress, hypoxia, and enhanced protein synthesis found in failing hearts potentially enhance ER stress in myocardium [5]. Okada and colleagues revealed that cardiac expressions of ER chaperones-GRP78 (glucose-regulated protein 78) and CHOP (CCAAT/enhancer-binding protein (C/EBP) homologous protein) were significantly increased at 1 and 4 weeks after transverse aortic constriction which induced cardiac hypertrophy and failure, respectively [6]. The endoplasmic reticulum, as the primary target in various disorders, determines the outcome of hypertrophic cardiomyocytes for restoring homeostasis or resulting in heart failure, through integrating the nucleus, mitochondria, and Golgi apparatus subcellular organelles reaction [7]. The adaptive and proapoptotic pathways of ER stress response play fundamental roles in the development and progression of cardiovascular diseases, including heart failure, ischemic heart diseases, and atherosclerosis. Thus, therapeutic interventions to regulate the gene expression of the target molecules for ER stress response and reduce ER stress should be the promising strategies for cardiovascular diseases treatment [8].

In Chinese medicine system, qi deficiency and blood stasis were the main pathogenesis of heart failure. Based on this theory Yiqi Huoxue method is widely used in clinical practice. Yiqi Huoxue recipe had been confirmed to be useful in treatment for heart failure in lab and clinical studies [9, 10]. It was reported that Yiqi Huoxue recipe improved heart function remarkably in patients with congestive heart failure, who were treated with the traditional Chinese medicine, on the bases of conventional therapy [10]. However, the mechanism of this effect was discussed little. In our study, we used the Yiqi Huoxue recipe (13 portions of* Astragalus*, 6 portions of* Angelica*, and 10 portion of* Ginseng*) as therapeutic drugs and the myocardial infarction (MI) rats as animal model, to study the molecular mechanisms of the cardioprotective effect of Yiqi Huoxue recipe in the MI rat model.

## 2. Materials and Methods

### 2.1. Chemicals and Drugs

Trizol was purchased from Invitrogen (CA, USA). Reverse transcription system (A3500) was purchased from Promega (WI, USA). The ab21685-100 antibody against GRP78 and the antibody against caspase-12 (Ab62484-100) were purchased from Abcam (Cambridge, UK). Antibody against GAPDH (Cs 2118) was provided from Cell Signaling Technology (Boston, US). The terminal deoxynucleotidyltransferase-mediated dUTP Nick End Labeling (TUNEL) kit was obtained from Wuhan Boster Biological Technology Ltd. (Wuhan, China). Other chemicals and reagents were of analytical grade.

*Preparation of Yiqi Huoxue Decoction.* Yiqi Huoxue Decoction consists of Astragalus,* Angelica*, and* Ginseng* in a 13 : 6 : 10 ratios. Slices of the herbs were provided by pharmaceutical preparation section, Dongzhimen hospital, Beijing, China. The medicinal herbs were extracted twice. Based on the ratios, Slices of those herbs were first boiled together in 6x volume of water for 0.5 h, and then the residue from first extraction was boiled in 8x volume of water for 25 min. Finally, the filtered solutions were combined and concentrated into the resulting aqueous extracts containing 1.2 g/mL raw herbs. Metoprolol Tartrate (MT) was purchased from AstraZeneca Company (Jiangsu, China).

### 2.2. Animals

Male Sprague-Dawley rats, weighing 190–210 g, were obtained from the laboratory of the Academy of Medical Sciences, Beijing, China (certificate number SCXK (Beijing) 2009–0007). The animals were fed by a standard laboratory diet and given free access to tap water. The cages were kept in a room with controlled temperature (22 ± 1°C), relative humidity (65–70%), and day/night cycle (12 : 12 light/dark). All animal procedures were carried out in accordance with the guidelines of the Institutional Animal Care and Use Committee of the University of Chinese Medicine, Beijing, China.

### 2.3. Myocardial Infarction

Rats were anesthetized with intraperitoneal sodium pentobarbital (Inactin Byk-Gulden, 50 mg/kg body weight) and then were artificially ventilated with a small animal volume-control ventilator (Harvard Apparatus; Holliston, MA) with a tidal volume of 1 mL at a rate of 100 cycles/min. Thoracotomy was then performed at the left third intercostal space; the heart was exposed via a small retractor. A 5-0 suture was placed in the anterior myocardium to occlude the left anterior descending artery (LAD). The thorax and the skin incision were closed with 2-0 sutures. The endotracheal tube was gently retracted after spontaneous breathing was restored. Sham operated animals were subjected to similar surgery, except that no ligature was placed [11]. Successful ligation was confirmed by ST segment elevation in postoperative ECG, compared with preoperative ones. All animals were given penicillin after the operation for three days to prevent infection. The surviving MI rats were divided randomly into three groups: MI (5 mL/kg/d normal saline (NS) by gavage), MI + MT (12 mg/kg/d MT by gavage), and MI + Yiqi Huoxue (5 mL/kg recipe by gavage). And the rats with sham operation were given 5 mL/kg/d NS by gavage. Treatments were given on the day following surgery once a day for 4 weeks.

### 2.4. Echocardiography

Four weeks after LAD ligation, transthoracic echocardiography was performed using an AloCa5000 system (Sino-Japanese joint) equipped with a 7–15 MHz real-time microvisualization scan head probe. Briefly, each rat was anesthetized with sodium pentobarbital (Inactin Byk-Gulden, 50 mg/kg body weight) and the chest was shaved. Then, echocardiographic parameters (left ventricular end-diastolic volume (LVDd), left ventricular end-systolic volume (LVDs), left ventricular ejection fraction (LVEF), and left ventricular fractional shortening (LVFS)) were measured.

### 2.5. Real-Time

RT-PCR Total RNA was extracted by TRIzol reagent according to the manufacturer's protocol (Invitrogen, Carlsbad, CA). For real-time PCR, reverse transcription was performed using 1 *μ*g RNA, AMV reverse transcriptase, and oligo (dT)_15_. PCR reactions were performed with the use of SYBR green master mix (ABI, Hercules, CA). The specific primer pairs are as follows: GRP78 forward 5′-CCACCAGGATGCAGACATTG-3′ and reward 5′-AGGGCCTCCACTTCCATAGA-3′ (100 bp); Caspase-12 forward 5′-CACTGCTGATACAGATGAGG-3′ and reward 5′-CCACTCTTGCCTACCTTCC-3′ (138 bp); GAPDH forward 5′-AGTTCAACGGCACAGTCAAG-3′ and reward 5′-TACTCAGCACCAGCATCACC-3′ (118 bp). Amplification was followed by melting curve analysis to verify the accuracy of the amplicon.

A negative control without cDNA was run with every PCR to assess the specificity of the reaction. PCR efficiency was between 90 and 110% for all primer sets. Analysis of data was performed through MxPro-Mx3000P software. Data were calculated as the change in cycle threshold (Δ*C*_*T*_) for the target gene relative to the Δ*C*_*T*_ for GAPDH (control gene) according to the procedures of Muller's et al. [12].

### 2.6. Western Blot

Heart tissues were homogenized on ice in RIPA buffer with a protease inhibitor (Complete, Roche). After centrifugation for 20 min at 4°C, the supernatant was used for Western blot analysis. Protein concentration was measured by BCA method (Bradford, 1976). 40–100 *μ*g proteins were loaded for Western blot analysis. The protein extract was transferred in sample buffer, loaded on 10% SDS-polyacrylamide gel, and blotted onto a nitrocellulose membrane with a wet blotting system. After being blocked for 1 h in Tris-buffered saline/Tween 20 (TBST) with 5% nonfat milk, the membranes were incubated with primary antibodies in blocking buffer (1 : 1000) at 4°C overnight. Then the membranes were incubated with peroxidase conjugated secondary antibody at a 1 : 6000 dilution at room temperature for 1 h. After being rinsed with TBST for 3 times, the proteins were detected by autoradiography on enhanced chemiluminescence (Super ECL Plus; Applygen Technologies, Beijing, China). Semiquantifications were performed with densitometric analysis using NIH ImageJ software.

### 2.7. Detection of Apoptosis in Heart Tissue

TUNEL stain was used to detect the apoptosis in heart tissues. Briefly, sections were deparaffinized and rehydrated and then pretreated with 20 *μ*g/mL proteinase K (37°C, 10 min). The sections were then incubated with TdT reaction mixture in a humidified chamber (37°C, 2 h), washed with PBS and blocked for 30 min at room temperature, and then incubated with anti-digoxigenin antibody at 37°C for 30 min. Subsequently, slides were incubated with streptavidin-biotin-peroxidase for 30 min. 3,3-Diaminobenzidine tetrahydrochloride was then added. Sections were counterstained with Mayer's hematoxylin. For negative staining controls, the TdT reaction mixture was omitted. All sections were then dehydrated and coverslipped. The percentage of apoptotic cells was determined; labeled nuclei were counted; and the results were expressed as percentage apoptotic nuclei of total nuclei in the heart tissue.

### 2.8. Statistical Analysis

Data are expressed as mean ± S.D. Statistical differences were evaluated by one-way ANOVA and then Newman-Student-Keuls test. A value of *P* < 0.05 was considered statistically significant.

## 3. Results

### 3.1. Yiqi Huoxue Recipe Attenuates Heart Function Injury and the Heart to Body Weight Ratio Increase in Myocardial Infarcted Rats

**Figure 1:**
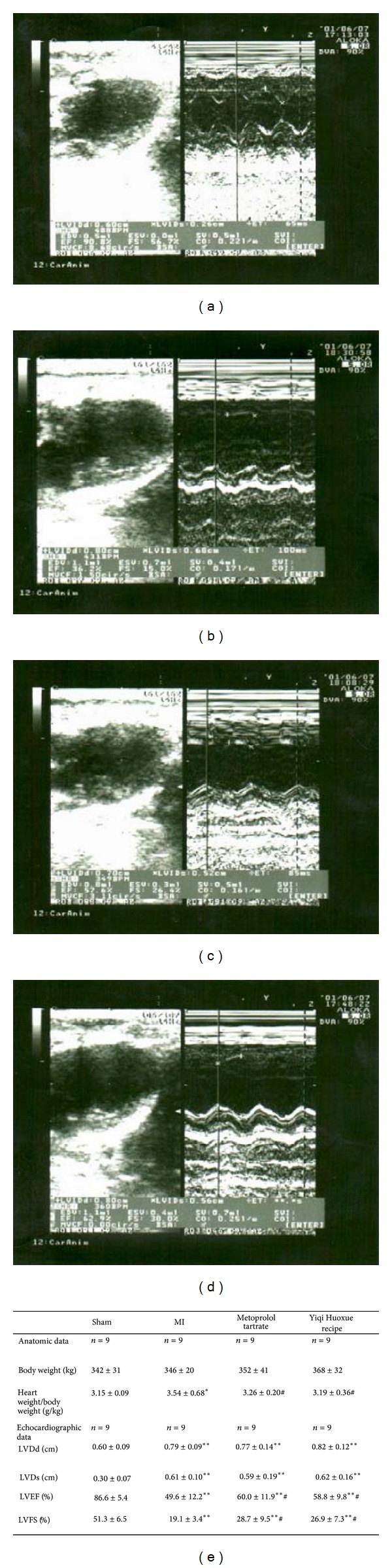
Yiqi Huoxue recipe attenuates heart function injury and attenuates the heart to body weight ratio in myocardial infarcted rats model. Rats were operated with myocardial infarction (MI) surgery and after 4 weeks of treatment, echocardiography assay was performed ((a) sham, (b) MI, (c) MI + Metoprolol Tartrate, (d) MI + Yiqi Huoxue recipe, and (e) anatomic and echocardiographic data for rats 4 weeks after myocardial infarction with different treatment). And after sacrificing the rats, the heart to body weight ratio was also calculated. In the rat myocardial infarction (MI) model, the heart to body weight ratio is increased in the untreated MI group at 4 weeks after MI. Yiqi Huoxue recipe and MT treatment diminished the effect (e). As to the echocardiographic data, the LVEF and LVFS showed the same trend (e). Values are mean ± SD (*n* = 9), LVEF, left ventricular ejection fraction; LVFS, left ventricular fractional shortening; LVDd, left ventricular end-diastolic volume; LVDs, left ventricular end-systolic volume. *P < 0.05, **P < 0.01 versus sham group. ^#^ *P* < 0.05 versus myocardial infarction group.

To determine the effect of the Yiqi Huoxue recipe on treatment of MI-induced heart failure in vivo, we induced MI in rats and detected the cardiac function by echocardiography assay and after sacrificing the rats we also measured the heart to body weight ratio. The result showed that a significant increase in the volume of left ventricle was demonstrated by the increase of LVDd and LVDs after 4 weeks of infarction (Figure 1(e)), which is attributed to the left ventricular dilatation. However, MT or Yiqi Huoxue recipe treatment had no significant effect on the MI-induced dilation. The result also demonstrated that the LVEF and LVFS were decreased significantly 4 weeks after MI surgery. Treatment with MT or Yiqi Huoxue recipe improved the cardiac function by increase in LVEF by 21% or 16.9% and in LVFS by 50.3% or 40.8%, respectively, compared with MI group (Figure 1(e)). However, MT or Yiqi Huoxue recipe showed no difference in the LVEF and LVFS improvements (*P* > 0.05). The heart to body weight ratio was increased in MI group rats compared with that in sham group rats (*P* < 0.05). The treatment with MT or Yiqi Huoxue recipe was observed to attenuate the increase in the heart to body weight ratio at 4 weeks after myocardial infarction surgery (*P* < 0.05) and showed no difference (*P* > 0.05) (Figure 1(e)).

### 3.2. Yiqi Huoxue Recipe Alleviates the Heart Tissue Histologic Injury in Myocardial Infarcted Rats

**Figure 2:**
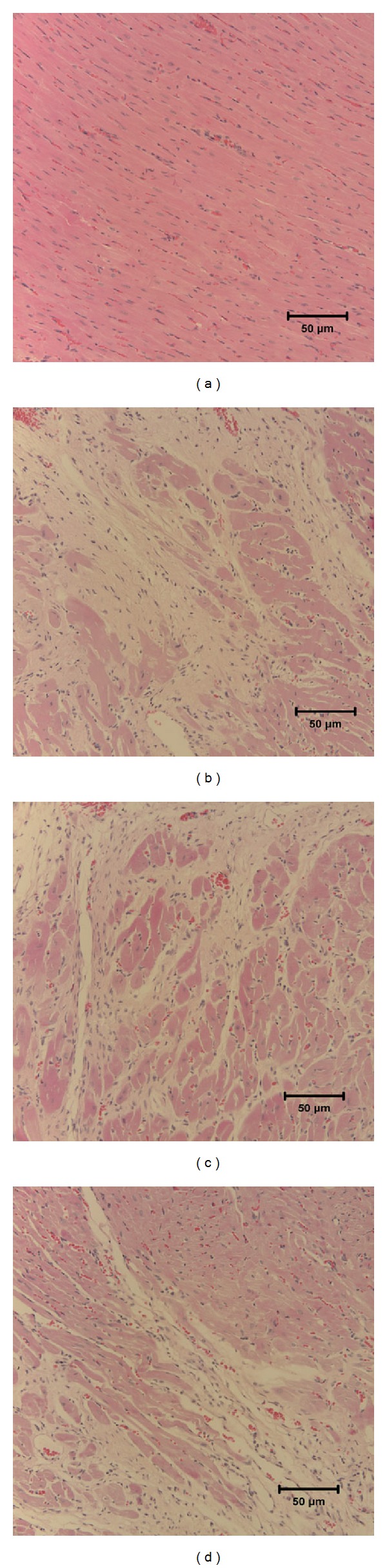
Microscopic observations (100x) of heart tissues. (a) Heart tissue of normal rat; (b) heart tissue of rat 4 weeks after MI; (c) heart tissue of rat treated with MT 4 weeks after MI; (d) heart tissue of rat treated with Yiqi Huoxue recipe 4 weeks after MI.

The microphotographs showed that in sham group myocardial fibers were arranged orderly, cytoplasmic staining was uniform, and nucleus boundaries were clear (Figure 2(a)). It was observed that in MI group the range of myocardial cells in heart tissue was in disorder and there is marked neutrophilic infiltration around the myocardial cells. Myocardiocyte disarrangement and fibrosis accretion were observed in MI group as well (Figure 2(b)). In rats group with 4 weeks of treatment with MT (Figure 2(c)) or Yiqi Huoxue recipe (Figure 2(d)) after MI surgery, myocardiocyte disarrangement, neutrophilic infiltration and fibrosis accretion were alleviated compared to rats with MI surgery only.

### 3.3. Yiqi Huoxue Recipe Attenuates Myocardial Apoptosis in MI Rats

**Figure 3:**
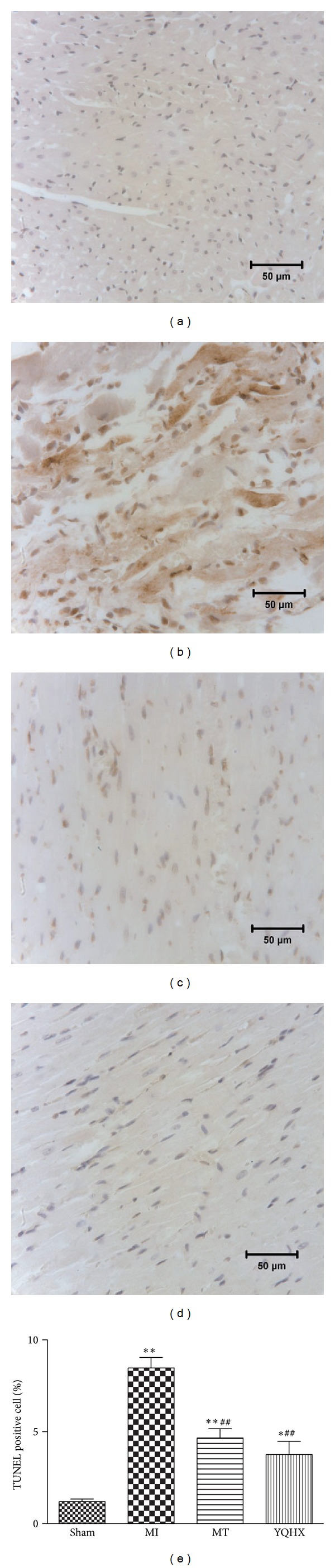
TUNEL staining of apoptosis in the border regions of heart sections from (a) sham rats, (b) MI rats, (c) MI + MT rats, and (d) MI + YQHX rats. Data are presented as the mean ± S.D. (e) Quantitative analysis of myocyte apoptosis in the border regions of rats after MI (*n* = 5). *P < 0.05, **P < 0.01 versus sham group. ^##^ *P* < 0.01 versus MI group.

Apoptosis has been suggested to be involved in the development of heart failure. To detect whether Yiqi Huoxue recipe protects heart function by apoptosis elimination, we performed the TUNEL assay. The results showed that LAD occlusion induced apoptosis in rats' heart tissue. As shown in Figure 3, at the border region, the ratio of TUNEL-positive myocardial cells to total myocardial cells was increased to 8.88%. In group treated with Yiqi Huoxue recipe or MT, the TUNEL-positive myocardial cell ratio was decreased to 4.12% or 4.52%, which was significantly lower than that in MI group (*P* < 0.01) and there was no difference between Yiqi Huoxue group and MT group.

### 3.4. Endoplasmic Reticulum Stress in the Heart of MI Rats Was Inhibited by Yiqi Huoxue Recipe

**Figure 4:**
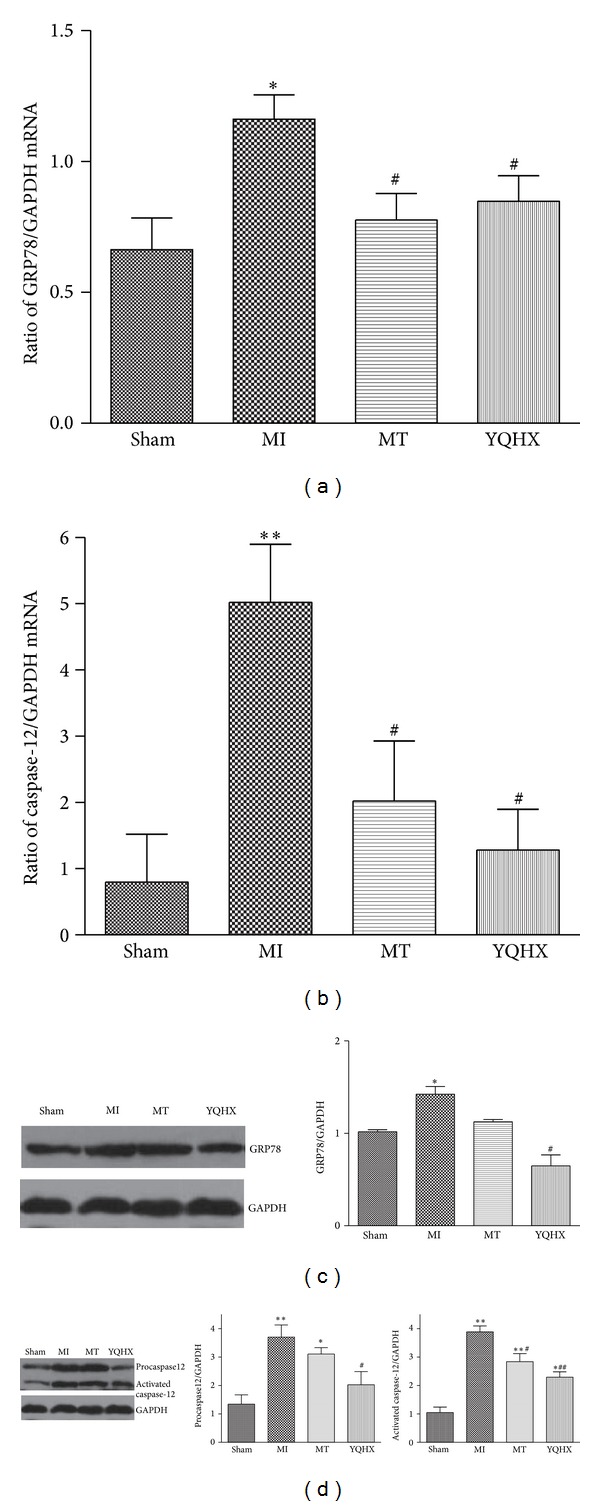
Expression of glucose response protein 78 (GRP78) and caspase-12 in mRNA level (a, b) and protein level (c, d) in heart tissues. Rats underwent MI surgery and after 4 weeks of treatment hearts were obtained. Heart tissues extracts were analyzed by immunoblotting by using antibodies against GRP78 and caspase-12. GAPDH was used as a loading control. Data are the mean ± SD (*n* = 3). *P < 0.05, **P < 0.01 versus sham group. ^#^ *P* < 0.05, ^##^ *P* < 0.01 versus myocardial infarction group.

Endoplasmic reticulum stress has been reported as an independent pathway leading to apoptosis and a new mechanism implicated in heart failure [5, 6]. To investigate the possible mechanism of heart protection of Yiqi Huoxue recipe, ERS related proteins such as GRP78 and caspase-12 were examined. Real-time PCR analysis of samples from heart tissue showed that GRP78 and caspase-12 mRNA levels were increased in MI rats hearts compared with the sham group (Figures 4(a) and 4(b)). When rats were treated with Yiqi Huoxue recipe or MT after LAD occlusion operation, GRP78 and caspase-12 mRNA levels in heart tissue were downregulated compared with those of MI rats without treatment (Figures 4(a) and 4(b)). Western blot showed that the protein levels of these two factors were increased in MI heart and Yiqi Huoxue recipe treatment can decrease the protein levels. And as to caspase-12, both the pro- and activated caspase-12 showed the same trend (Figures 4(c) and 4(d)).

## 4. Discussion

Heart failure, as the end point of most heart diseases, is a major public health problem with high morbidity and mortality, frequent hospitalizations, and a major cost burden on the community. Up to now, researches on the mechanisms and therapies of heart failure have made much progress [13, 14]. It is well known that activated neurohormonal system is one of the most important pathophysiologic factors. In particular, the sympathetic nervous system (SNS) and renin-angiotensin-aldosterone system (RAAS) are the main reasons which contribute to the progression and pathophysiology of cardiac dysfunction. Thus, *β*-blockers, angiotensin-converting enzyme (ACE) inhibitors, aldosterone-receptor blockers, and angiotensin-receptor blockers (ARB) have all been found in major trials to confer morbidity and mortality benefits and thus are the mainstays of current treatment as recommended by guideline authorities. However, due to the complexity and severity of the symptom, the mortality remains unacceptably high at around 8–12% annually [15]. Therefore, novel and effective drugs that show few side-effects are needed [16, 17].

Traditional Chinese medicine has been used for a long time and formed a theory of diagnosis and treatment of heart failure [18–21]. Yiqi Huoxue recipe used in the current experiment is composed of* Astragalus*,* Angelica*, and* Ginseng*.* Astragalus*, the main ingredient of our recipe, is one of the most commonly used elements in traditional Chinese medicine for chronic heart failure in China. Modern pharmacological research has shown that* Astragalus* injection can enhance myocardial contractility, improve circulation, protect myocardial cells, and regulate immunity [22].* Angelica*, another component of the recipe, contains organic acids, which has been proved to have the function of antimyocardial ischemia by stabilizing the cell member system [23, 24]. The third component of recipe use in our study,* Ginseng*, is widely used in traditional Chinese medicine having cardiovascular benefits usually used in treatment of heart disease [25]. And emerging evidence also suggests that* Ginseng* attenuates myocardial hypertrophy, thus blunting the remodeling and heart failure processes [26].

In current study, we used recipe composed of the above three traditional Chinese herbs to deal with the heart failure development after LAD occlusion surgery in rat model, to explore the mechanisms underlying its protective effects.

The result demonstrated that rats developed heart failure 4 weeks after LAD occlusion surgery and the recipe or MT attenuated the heart function damage. Ratio of heart to body weight was increased in MI group and the increase was eliminated in recipe and MT groups. The recipe showed more effectiveness in the general condition of the model rats compared with other groups. The rats' body weight in Chinese medicine group took advantage over that in MT group. Due to the individual difference of rats' response to Chinese medicine, the difference between MT and Chinese medicine group is not obvious after statistical analysis. However, the improvement tendency shown in Chinese medicine group suggests the Chinese medicine superiority in survival benefit, which is superior to the single-target therapy with western medicine. To discuss the apoptosis in the mechanism of heart protection of the recipe, rats underwent LAD occlusion surgery were sacrificed 4 weeks later and TUNEL assay was performed in the heart sections. The apoptosis was increased obviously in MI group and the recipe could inhibit the increase partially. These results suggest that, in our experiment, the recipe could improve the injured heart function through eliminating myocyte apoptosis. Since the ER stress response pathway is an emerging compartment involved in the apoptosis, we further detected the ER stress marker-GRP78 and ER stress-specific apoptosis inducing protein caspase-12. Results showed that GRP78 and caspase-12 were upregulated in heart tissue of MI group rats. Treatment with Yiqi Huoxue recipe significantly attenuated upregulated GRP78 protein expression and caspase-12 activation induced in hear failure. This suggests that a reduced ER stress response is involved in the protective effect of Yiqi Huoxue recipe against heart failure progression.

The ER is an organelle that has essential roles in multiple cellular processes including intracellular calcium homeostasis, protein secretion, and lipid biosynthesis [27]. Multiple disturbances in cellular redox regulation or calcium regulation caused by hypoxia, oxidants, glucose deprivation, or viral infection can cause accumulation of unfolded proteins in the ER, triggering an evolutionarily conserved response termed as the unfolded protein response (UPR) [28]. UPR induced by ER stress can lead to adaptation by expression of genes that are capable of enhancing the protein folding capacity of the ER and genes for ER-assisted degradation. If the adaptive mechanisms fail to compensate, the cell death is induced, typically by apoptosis. ER stress leads to apoptosis by mitochondria-dependent and -independent pathways. ER disturbances can lead to mitochondrial disruption and ER stress has been determined to signal apoptosis through a mitochondrial-dependent pathway. The mitochondrial-independent pathway is thought to occur through the initiator caspase-12 [29]. All the information suggests that ER-specific apoptosis pathway is an important apoptosis pathway [4]. Recent researches have shown the existence of spliced XBP1 (X-box-binding protein-1) and markedly increased GRP78 expression, suggesting that UPR activation is associated with the pathophysiology of heart failure [6, 30]. In the current study, we also detected increased GRP78 expression and caspase-12 in heart tissue of rats with MI-induced heart failure. And the result suggests that ER stress may be an important mechanism involved in Yiqi Huoxue recipe myocardial cells protection. The components of Yiqi Huoxue recipe such as* Astragalus*,* Angelica*, and* Ginseng* had been confirmed to have antioxidant function and can remove free radicals [31]. Reactive oxygen species or oxidative stress, as the main mechanism included in heart failure progression, has been reported to induce ER stress [32]. Taken together, all the information showed that reactive oxygen species may be a link from myocardial cells protection of Yiqi Huoxue recipe to ER stress inhibition in the treatment of heart failure. However, further study is required to investigate the mechanism of the Yiqi Huoxue recipe in protection of myocardial cells.

In conclusion, the current research demonstrated that Yiqi Huoxue recipe, composed of* Astragalus*,* Angelica*, and* Ginseng*, can improve the injured heart function in vivo. And it can inhibit the apoptosis and ER stress response pathway in MI-induced heart failure development. All the results suggest that Yiqi Huoxue recipe improves the injured heart function maybe through inhibition of ER stress response pathway and ER stress response pathway is a promising target in therapy for MI-induced heart failure development.

## Abbreviations

ACE: Angiotensin-converting enzyme
ARB: Angiotensin-receptor blockers
CHOP: CCAAT/enhancer-binding protein homologous protein
CAD: Coronary artery disease
ER: Endoplasmic reticulum
GRP78: Glucose-regulated protein 78
LAD: Left anterior descending artery
LVEF: Left ventricular ejection fraction
LVDd: Left ventricular end-diastolic volume
LVDs: Left ventricular end-systolic volume
LVFS: Left ventricular fractional shortening
MT: Metoprolol Tartrate
MI: Myocardial infarction
NS: Normal saline
RAAS: Renin-angiotensin-aldosterone system
SNS: Sympathetic nervous system
UPR: Unfolded protein response
XBP1: X-box-binding protein-1.

## References

[1] Tarride JE, Lim M, DesMeules M (2009). A review of the cost of cardiovascular disease. *Canadian Journal of Cardiology*.

[2] Ottaviani G, Lavezzi AM, Rossi L, Matturri L (1999). Proliferating cell nuclear antigen (PCNA) and apoptosis in hyperacute and acute myocardial infarction. *European Journal of Histochemistry*.

[3] Olivetti G, Quaini F, Sala R (1996). Acute myocardial infarction in humans is associated with activation of programmed myocyte cell death in the surviving portion of the heart. *Journal of Molecular and Cellular Cardiology*.

[4] Rao RV, Ellerby HM, Bredesen DE (2004). Coupling endoplasmic reticulum stress to the cell death program. *Cell Death and Differentiation*.

[5] Toth A, Nickson P, Mandl A, Bannister ML, Toth K, Erhardt P (2007). Endoplasmic reticulum stress as a novel therapeutic target in heart diseases. *Cardiovascular and Hematological Disorders—Drug Targets*.

[6] Okada KI, Minamino T, Tsukamoto Y (2004). Prolonged endoplasmic reticulum stress in hypertrophic and failing heart after aortic constriction: possible contribution of endoplasmic reticulum stress to cardiac myocyte apoptosis. *Circulation*.

[7] Minamino T, Kitakaze M (2010). ER stress in cardiovascular disease. *Journal of Molecular and Cellular Cardiology*.

[8] Minamino T, Komuro I, Kitakaze M (2010). Endoplasmic reticulum stress as a therapeutic target in cardiovascular disease. *Circulation Research*.

[9] Li H, Zhang Y, Ma J (2011). [Effects of yiqi huoxue compound combined with exercise therapy on MMP-1 and collagen type III expressions of cardiac muscle in chronic heart failure rats]. *Zhongguo Zhong Xi Yi Jie He Za Zhi*.

[10] Li D-P, Chen Q, Yi L (2006). Effects of yiqi huoxue method on cardiac function in patients with congestive heart failure. *Zhongguo Zhong Xi Yi Jie He Za Zhi*.

[11] Wu A, Zhai J, Zhang D (2013). Effect of wenxin granule on ventricular remodeling and myocardial apoptosis in rats with myocardial infarction. *Evidence-Based Complementary and Alternative Medicine*.

[12] Muller PY, Janovjak H, Miserez AR, Dobbie Z (2002). Processing of gene expression data generated by quantitative real-time RT-PCR. *BioTechniques*.

[13] Zile MR, Gaasch WH (2011). Abnormal calcium homeostasis: one mechanism in diastolic heart failure. *Journal of the American College of Cardiology*.

[14] Gu R, Lu W, Xie J, Bai J, Xu B (2011). Renalase deficiency in heart failure model of rats-A potential mechanism underlying circulating norepinephrine accumulation. *PLoS ONE*.

[15] Sata Y, Krum H (2010). The future of pharmacological therapy for heart failure. *Circulation Journal*.

[16] Wang J, Xiong XJ (2012). Current situation and perspectives of clinical study in integrative medicine in China. *Evidence-Based Complementary and Alternative Medicine*.

[17] Xiong XJ, Yang XC, Liu YM, Zhang Y, Wang PQ, Wang J (2013). Chinese herbal formulas for treating hypertension in traditional Chinese medicine: perspective of modern science. *Hypertension Research*.

[18] Fu S, Zhang J, Menniti-Ippolito F (2011). Huangqi injection (a traditional chinese patent medicine) for chronic heart failure: a systematic review. *PLoS ONE*.

[19] Chen CX, Gao JP, Wu Q, Guo J, Gu WL (2010). Progress in treatment of chronic heart failure in Western medicine and treatment strategies in traditional Chinese medicine. *Zhong Xi Yi Jie He Xue Bao*.

[20] Wang J, Wang PQ, Xiong XJ (2012). Current situation and re-understanding of syndrome and formula syndrome in Chinese medicine. *Internal Medicine*.

[21] Chen J, Wu G, Li S (2007). Shengmai (a traditional Chinese herbal medicine) for heart failure. *Cochrane Database of Systematic Reviews*.

[22] Zhang JG, Gao DS, Wei GH (2002). Clinical study on effect of Astragalus injection on left ventricular remodeling and left ventricular function in patients with acute myocardial infarction. *Zhongguo Zhong Xi Yi Jie He Za Zhi*.

[23] Wang XM, Li YD (2009). Research progress of effective component and pharmacological effect of Radix Angelicae Sinensis. *Gansu Journal of Traditional Chinese Medicine*.

[24] Yu L, Li JZ, Wang HY (2001). Progress in the study of the treatment of nephropathy with Astragalus and Angelica and their therapeutic mechanism. *Zhongguo Zhong Xi Yi Jie He Za Zhi*.

[25] Zheng SD, Wu HJ, Wu DL (2012). Roles and mechanisms of ginseng in protecting heart. *Chinese Journal of Integrative Medicine*.

[26] Karmazyn M, Moey M, Gan XT (2011). Therapeutic potential of ginseng in the management of cardiovascular disorders. *Drugs*.

[27] Anelli T, Sitia R (2008). Protein quality control in the early secretory pathway. *The EMBO Journal*.

[28] Ron D, Walter P (2007). Signal integration in the endoplasmic reticulum unfolded protein response. *Nature Reviews Molecular Cell Biology*.

[29] Nakagawa T, Zhu H, Morishima N (2000). Caspase-12 mediates endoplasmic-reticulum-specific apoptosis and cytotoxicity by amyloid-*β*. *Nature*.

[30] Sawada T, Minamino T, Fu HY (2010). X-box binding protein 1 regulates brain natriuretic peptide through a novel AP1/CRE-like element in cardiomyocytes. *Journal of Molecular and Cellular Cardiology*.

[31] Li HT, Su DH, Shang T, Pan WG, Gao P (2008). Antioxidant activity of the essential oils and Aqueous extracts obtained from five chinese herbal medicines. *Natural Product Research Development*.

[32] Hanada S, Harada M, Kumemura H (2007). Oxidative stress induces the endoplasmic reticulum stress and facilitates inclusion formation in cultured cells. *Journal of Hepatology*.

